# Utilization of *Phyllanthus emblica fruit* *stone* as a Potential Biomaterial for Sustainable Remediation of Lead and Cadmium Ions from Aqueous Solutions

**DOI:** 10.3390/molecules27103355

**Published:** 2022-05-23

**Authors:** Sarita Kushwaha, Suhas, Monika Chaudhary, Inderjeet Tyagi, Rakesh Bhutiani, Joanna Goscianska, Jahangeer Ahmed, Manila, Shubham Chaudhary

**Affiliations:** 1 Department of Chemistry, Gurukula Kangri (Deemed to be University), Haridwar 249404, India; saritakushwaha31@gmail.com (S.K.); monikachoudry@gmail.com (M.C.); drmanilache@gmail.com (M.); shubhamchaudhary89@yahoo.com (S.C.); 2 Centre for DNA Taxonomy, Molecular Systematics Division, Zoological Survey of India, Kolkata 700053, India; indertyagi011@gmail.com; 3 Department of Zoology & Environmental Sciences, Gurukula Kangri (Deemed to be University), Haridwar 249404, India; rbhutiani@gmail.com; 4 Department of Chemical Technology, Faculty of Chemistry, Adam Mickiewicz University in Poznań, Uniwersytetu Poznańskiego 8, 61-614 Poznań, Poland; asiagosc@amu.edu.pl; 5 Department of Chemistry, College of Science, King Saud University, P.O. Box 2455, Riyadh 11451, Saudi Arabia; jahmed@ksu.edu.sa

**Keywords:** *Phyllanthus emblica* *fruit* *stone*, lead, cadmium, biosorption, pseudo-second order kinetic model, adsorption isotherm

## Abstract

In the present work, an effort has been made to utilize *Phyllanthus emblica* (PE) fruit stone as a potential biomaterial for the sustainable remediation of noxious heavy metals viz. Pb(II) and Cd(II) from the aqueous solution using adsorption methodology. Further, to elucidate the adsorption potential of *Phyllanthus emblica fruit stone* (*PEFS*), effective parameters, such as contact time, initial metal concentration, temperature, etc., were investigated and optimized using a simple batch adsorption method. It was observed that 80% removal for both the heavy metal ions was carried out within 60 min of contact time at an optimized pH 6. Moreover, the thermodynamic parameters results indicated that the adsorption process in the present study was endothermic, spontaneous, and feasible in nature. The positive value of entropy further reflects the high adsorbent–adsorbate interaction. Thus, based on the findings obtained, it can be concluded that the biosorbent may be considered a potential material for the remediation of these noxious impurities and can further be applied or extrapolated to other impurities.

## 1. Introduction

Water, being the most essential element and basic requirement of the living creatures on Earth, constitutes >70% of the entire earth’s surface, out of which only 0.002% is considered appropriate for human consumption [1]. Due to rapid urbanization and industrialization, it is a very tough challenge for us to prevent this precious resource from getting polluted [2]. Pollutants containing different heavy metal ions from various sources, such as oil refining, tannery, paints, batteries, electrical, metal plating, pigments and chemical manufacturing were discharged into our water resources [3,4]. Other than the external heavy metals (HMs) polluting sources, they are present as natural constituents with varying concentrations in the Earth’s crust [5]. These heavy metal ions get accumulated as a toxicant in the aquatic biota and possess a severe detrimental adverse impact on human and faunal health. The HMs are considered persistent environmental pollutants due to their availability for several years in the complex food web network [5]. Among these HMs, in the present work, we consider Pb(II) and Cd(II) as model pollutants as they are broadly distributed across the environment and thereby develop chances for an average person to come into contact with them. Literary pieces of evidence supported the fact that chronic exposure of Pb(II) in humans causes reproductive and neurological disorders and also possesses certain genotoxic as well as carcinogenic effects on human health. On the other hand, chronic exposure of Cd(II) causes different malfunctions, such as lesions, pulmonary cancer, nephrological and osteological disorders, etc. [6,7,8].

Keeping in view the severe toxicity of these metal ions, the US Environmental Protection Agency (USEPA) prescribed 1.0 mg L^−1^ as the maximum permissible concentration of Pb in industrial wastewater and 0.015 mg L^−1^ in drinking water, while the Indian agency, the Central Pollution Control Board (CPCB) limits it to 0.1 mg L^−1^ as the maximum permissible concentration in the inland surface water. The Bureau of Indian Standards recommended 0.01 mg L^−1^ as the maximum admissible limit of Pb(II) concentration in drinking water. Moreover, the environmental and health agencies, such as USEPA, WHO and CPCB, prescribe 0.005, 0.003 and 0.003 mg L^−1^, respectively, as the maximum permissible limit of Cd(II) in the drinking water beyond this limit, with continuous exposure, may lead to several health hazards, as discussed in the preceding paragraphs.

Thus, based on the properties of HMs, such as their ubiquitous nature; long-term stability; high toxicity, even in trace concentrations; a tendency to bio-accumulate, etc., led to heavy metal pollution to become a relatively scorching issue that needs to be addressed with immediate effect regarding the advent of new technologies. To date, different techniques [9,10], such as reverse osmosis, coagulation/flocculation, ion exchange, precipitation, biosorption, etc., have been used for the removal of metals, and each has certain advantages as well as disadvantages. However, it is worthwhile to note that out of these, the biosorption process through accumulation—with the help of biological material—helps in the restoration or disposal of the pollutants in an acceptable range by removing them from water [11]. Further, the biosorption process has several advantages, such as being economical, sludge free, providing the restoration of sorbate, and being highly selective for pollutants and their removal efficiency [12,13].

A number of biomasses [8,14,15,16,17,18,19,20,21,22,23] have been used for the biosorption process viz., wheat straw, apple pomace, rice husk, jujube shell, orange peels, *Orbignya speciosa*, *Phyllanthus emblica* bark, *Phyllanthus emblica* leaf powder, etc. Owing to their low market value and abundance, the natural biomass material may act as a promising choice for biosorption. *Phyllanthus emblica fruit stone* (*PEFS*), an important lignocellulosic carbonaceous biomass, is available in significant amounts in India. The fruit of *Phyllanthus emblica* (*PEF*) has many medicinal values [24] and is utilized for a variety of long-established curing of diseases in India. Furthermore, PEF [25,26] is also used in making traditional edible foodstuffs, such as jams, jellies, tarts, chutneys, etc., and after removing the pulp from *emblica* fruit, the remaining fruit stone is more or less a waste. Due to the large use of PEF, a massive number of fruit stones are disposed of as waste per year, which are not perilous and may be utilized for other purposes. It is worth mentioning, however, that except few marginal applications, *PEFS* does not have any major utility in industry, hence, they can be used for the removal of harmful metals.

Hence, in the present work, we used a biosorbent *PEFS* and applied it for the sustainable remediation of noxious Pb(II) and Cd(II) from aqueous solutions. To be specific, the significant objectives of the current work are: (1) To utilize biomass *PEFS* as a biosorbent and to explore its potential in remediation of Pb(II) and Cd(II); (2) to elucidate the impact of effective parameters, such as contact time, initial metal ion concentration, pH and temperature on the remediation of noxious Pb(II) and Cd(II) from the aqueous solutions; (3) to explore the thermodynamics, kinetics and isotherm model that suited the adsorptive removal of selected model pollutants.

## 2. Results and Discussion

### 2.1. Effect of Contact Time

**Figure 1:**
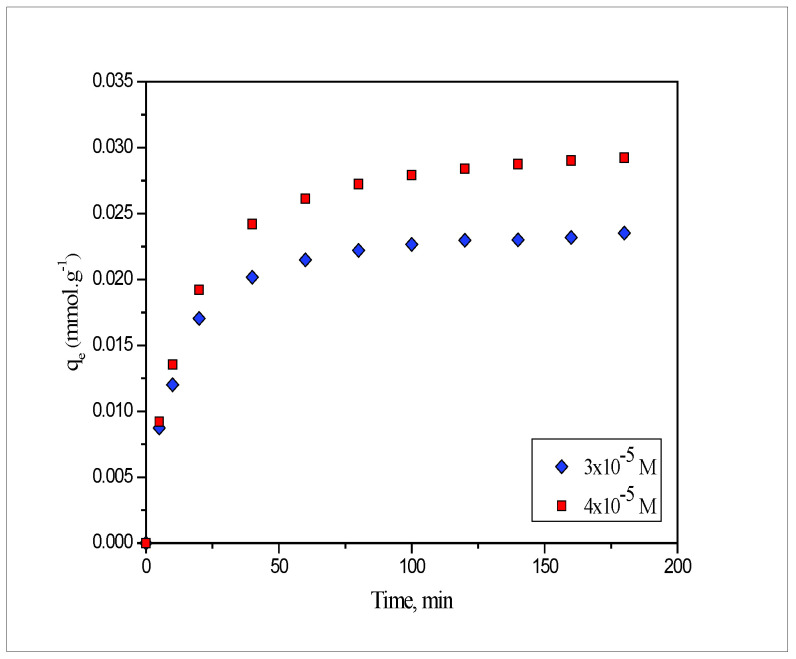
Effect of contact time and initial concentration on the biosorption of (a) Pb(II) onto *PEFS* at 25 °C.

To determine the appropriate contact time for lead and cadmium metal ion removal by PEFS, the biosorption capacity for Pb(II) and Cd(II) was measured as a function of time at two different concentrations. The results acquired at 25 °C for Pb(II), are shown in Figure 1 and a similar trend is observed for Cd(II), therefore, not shown here. From Figure 1, it was observed that initially, the uptake of adsorbate species on the PEFS biosorbent was fast, which later becomes slow until equilibrium was achieved. The maximum amount (80%) of lead and cadmium metal ion was removed in less than 60 min and thereafter, equilibrium was achieved in 120 min. Thus, for all equilibrium adsorption studies, the contact time was kept at 180 min and after this, no appreciable increase in the metal amount adsorbed was observed. This might be accredited to the rapid utilization of the freely accessible adsorbing sites [27] on the biosorbent surface.

### 2.2. Effect of Initial Metal Ion Concentration

The effect of variation in the initial concentration of Pb(II) and Cd(II) on the biosorption process of the *PEFS* was also elucidated. The amount of lead and cadmium metal ions adsorbed on the *PEFS* at two different concentrations, 3 × 10^−5^ and 4 × 10^−5^ M for Pb(II), and 2 × 10^−5^ and 3 × 10^−5^ M for Cd(II), was studied. The results obtained for Pb(II) are presented in Figure 1. Experimental observation from the results revealed that with the increase of the initial metal ion concentration, the amount of Pb(II) and Cd(II) adsorbed per unit mass of the *PEFS* increased; however, the percentage of the biosorption was found to decrease. This may be due to the fact that at low metal ion concentrations, all the metal ions present in the aqueous solution might interact with the binding sites present on the biosorbent, thus, the percentage of the biosorption was greater in contrast to the higher concentration [28].

### 2.3. Effect of pH

**Figure 2:**
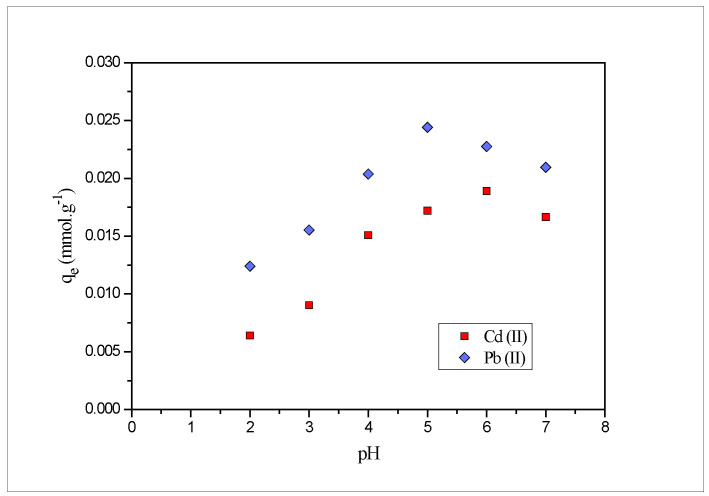
Effect of pH on the biosorption of Pb(II) and Cd(II) onto *PEFS* at 3 × 10^−5^ M.

The pH level is one of the important key parameters that influence the sorption efficiency by affecting the solubility of the metal and the total charge of the biosorbent functional group [29]. In this study, the effect was investigated within the pH range (2–7) keeping other parameters constant (contact time: 180 min, adsorbent dose: 0.01 g/10 mL, temperature: 25 °C, metal ion concentration: 3 × 10^−5^ M). Experiments were not conducted at higher pH as lead as well as cadmium metal ions were hydrolyzed and precipitated in an alkaline medium instead of their adsorption [30]. The amount of the Pb(II) and Cd(II) removed at different pH is presented in Figure 2. At lower pH [31], removal of the lead and cadmium was inhibited due to the competition between the metal ions and hydrogen ions for adsorption sites present on the biosorbent surface, thus making it inaccessible for the metal binding [32]. Whereas, at higher pH, the lower amount of protons in the solution results in the reduced competition with the metal ions to be biosorbed onto the surface of *PEFS*. This fact is also supported by the point of zero charge (pHpzc = 3.4) of *PEFS*. More cations, namely Pb(II) and Cd(II) are adsorbed onto the surface of *PEFS* at pH > pHpzc, as the surface of the *PEFS* becomes negatively charged, thereby enabling for the biosorption of positively charged metal ions due to the electrostatic attraction and less competition with protons.

The amount of lead metal ion adsorbed increased on increasing the pH from 2 to 5 and further removal decreased with an increase in the pH from 5 to 7. The maximum removal efficiency was obtained at pH 5; hence, all further experimental studies were carried out at pH 5. Similarly, for cadmium metal ions, the amount adsorbed increased from pH 2–6 and decreased further from 6–7, the maximum amount adsorbed at pH 6, hence, all the experiments were further carried out at pH 6.

### 2.4. Adsrption Isotherms

**Figure 3:**
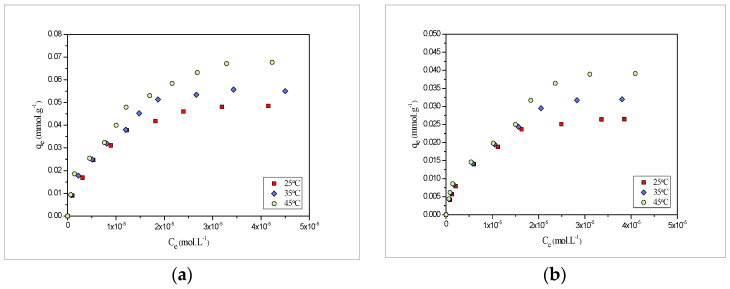
Adsorption isotherms of (**a**) Pb(II) and (**b**) Cd(II) onto *PEFS* at 25, 35 and 45 °C.

Adsorption isotherm defines the relation between the quantity of the adsorbate adsorbed by the biosorbent material and the concentration of the adsorbate remaining in the solution after the system attained the equilibrium at a constant temperature. In order to evaluate the effectiveness of the *PEFS* for the removal of the metal ion, the equilibrium sorption of the Pb(II) and Cd(II) was studied as a function of the concentration and the adsorption isotherm obtained is shown in Figure 3.

The experimental values obtained at 25, 35 and 45 °C were found to be 0.048, 0.055 and 0.068 mmol·g^−1^ for Pb(II) and 0.026, 0.032 and 0.039 mmol·g^−1^ for Cd(II), respectively. Comparative results clearly show that Pb(II) removal is more than compared to Cd(II). 

Further, the adsorption isotherm models, such as Langmuir and Freundlich, were applied to optimize the sorption process isotherm data. The former isotherm model, i.e., the Langmuir isotherm model, was effectively used to reveal the monolayer sorption onto the fixed number of identical sites, and is represented using the equation mentioned below [33]:

(1) $$\frac{1}{q_{e\;}}=\frac{1}{q_{m\;}}+\frac{1}{q_{m}{\mathrm{bC}}_{e}}$$

where

The amount of model pollutant adsorbed at equilibrium is represented by q_e_;

The maximum monolayer adsorption capacity is measured by q_m_;

Langmuir equilibrium constant is represented by b;

Equilibrium concentration is represented as C_e_.

**Figure 4:**
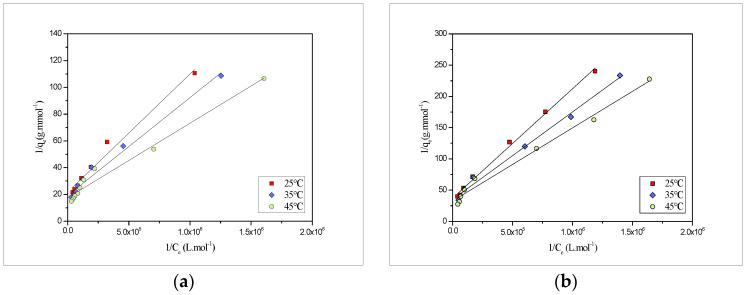
Langmuir adsorption isotherms of onto *PEFS* at 25, 35 and 45 °C for (**a**) Pb(II) and (**b**) Cd(II).

The Langmuir plots, i.e., 1/q_e_ and 1/C_e_ obtained for the biosorption of model pollutants Pb(II) and Cd(II) is shown in Figure 4. 

Further, the values of the maximum monolayer adsorption (q_m_) and Langmuir constant (b) were measured from the intercept and slope of the plot and are compiled in Table 1. 

**Figure 5:**
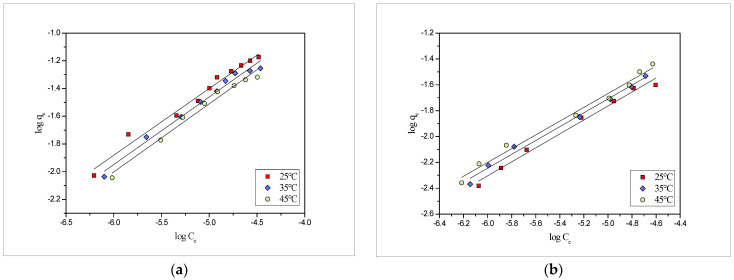
Freundlich adsorption isotherms of onto *PEFS* at 25, 35 and 45°C for (**a**) Pb(II) and (**b**) Cd(II).

On the other hand, the Freundlich adsorption isotherm model was used to reveal the multilayer adsorption and was based on the theory of the multilayer sorption process [34]. It was represented linearly using the equation mentioned below:

logq_e_ = log k_F_ + (1/n) log C_e_ (2)

where q_e_ is the amount of the metal adsorbed at the equilibrium concentration C_e_, k_F_ (mmol·g^−1^) (L·mol^−1^)^1/n^ and n (unitless) represented constants linked with adsorption capacity and adsorption intensity, respectively. Freundlich plots between log C_e_ and log q_e_ for the adsorption of the Pb(II) and Cd(II) are given in Figure 5. Values obtained for all the isotherm constants and correlation coefficients are tabulated in Table 1. From the tabulated values (Table 1), it can be observed that both the Langmuir and Freundlich models follow well to the experimental data in both cases Pb(II), as well as Cd(II). The results of the adsorption process show that the adsorption of the lead is higher than the cadmium on the *PEFS*. This may be due to the fact that ionic radius plays an important role in the adsorption of metal on the biosorbent surface. Though the lead and cadmium have the same valency, the ionic radius of the lead was larger than the cadmium and, thus, owing to the smaller size and higher charge densities, the cadmium ion will attract more water molecules and form larger hydrated ion in comparison to lead. Therefore, the access of the cadmium to the biosorbent surface will be less [35].

The shape of the isotherm may also indicate the favorability of the adsorption, which can be discussed by the parameter ‘R_L_’ [36]. It is termed an equilibrium parameter or separating factor and can be calculated using the equation mentioned below:

(3) $$R_{L}=\frac{1}{1+{b\;C}_{0}}$$

where the initial concentration is represented by C_0_ and the Langmuir constant is represented by b. Further, values of R_L_ between 0 and 1 indicate a favorable adsorption isotherm [36].

### 2.5. Characterisation of PEFS and Mechanism

The work reported here is a further study of the biosorbent reported elsewhere [37], and some of the properties of the *PEFS*, as discussed elsewhere, are recapitulated in Table 2. Table 2 shows that the *PEFS* has a considerable carbon content (46.46%), whereas, among the inorganic contents (Table 2), calcium and potassium are found in large quantities as compared to the other inorganic elements. Pb and Cd were found to be nearly absent in the material. The stability of the *PEFS* in water was also tested, and it was found that the adsorbent does not dissolve in water, which makes it a perfect biosorbent. Furthermore, the functional groups observed on the surface of the *PEFS* were also determined with the help of Fourier transform infrared spectroscopy (FTIR) (Table 2). Besides this, the surface area of *PEFS* was found to be below, and the material was non-porous in nature, as discussed elsewhere [37]. Moreover, field emission scanning electron microscopy (FE-SEM) also favors this observation, as the surface of *PEFS* is compact in nature and non-porous.

The biosorption behavior of metal ions on the *PEFS* is a plausible mechanism with physical adsorption, surface complexation, electrostatic attraction and ion exchange as conventional pathways in explaining the removal of Pb(II) and Cd(II) by *PEFS* [38,39]. The FTIR analysis (Table 2) of the *PEFS* shows that the main functional groups are -C-H, -OH, C=O, C–O, methoxy and carboxylate anion. Out of these, the carboxyl and hydroxyl group plays an important role in the removal of metal ions on the *PEFS* mechanism. Owing to the presence of these groups on the surface, *PEFS* attracts positively charged metal ions via electrostatic interactions and ion exchange methods. Finally, after these interactions, with the entrapping of metal ions in the *PEFS* surface, the complexation occurs, which is hypothesized via -COO and -OH interactions with Pb(II) and Cd(II) [38,39,40,41]. These functional groups, mainly the carboxyl and hydroxyl groups, get deprotonated at pH > pHpzc and the metal ions (Pb^2+^ and Cd^2+^) form complexes with the anionic form of these groups, resulting in the enhanced biosorption of the *PEFS* surface [32,42]. Therefore, it can be demonstrated that biosorption may occur through metal complexation [43,44] with the functional groups, such as hydroxyl and carboxyl present on the *PEFS* surface [32].

### 2.6. Effect of Temperature and Thermodynamic Parameters

**Figure 6:**
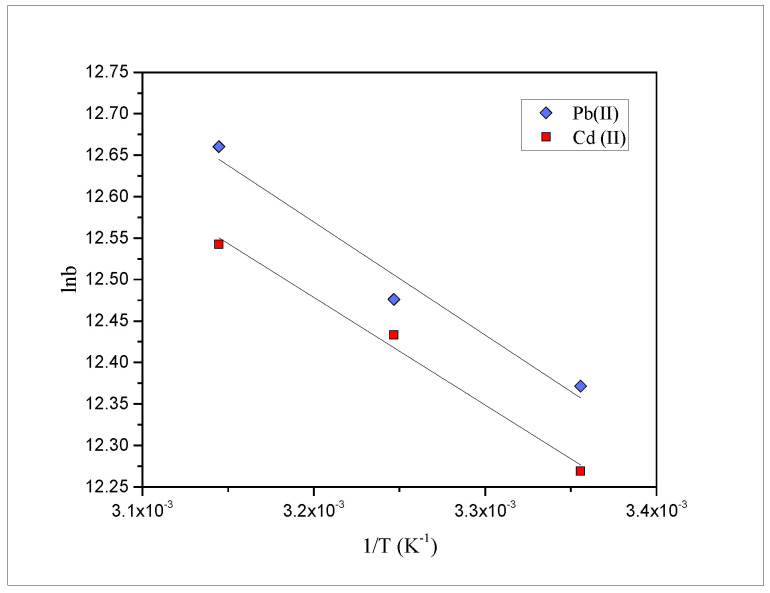
The van’t Hoff plots for the biosorption of Pb(II) and Cd(II) onto *PEFS*.

To elucidate the effect of temperature, three different temperatures i.e., 25, 35 and 45 °C, were selected, and the findings obtained are shown in Figure 3. Results obtained indicated the fact that with the increase in temperature, the biosorption also increases. Thus, the process may be endothermic in nature. Further, the thermodynamic parameters, such as ∆G° (kJ mol^−1^), ΔH° (kJ mol^−1^) and ΔS° (J mol^−1^ K^−1^) were also measured using the below-mentioned equations:

(4) ΔG°=−RTln(b)

(5) $$\mathrm{lnb}=-\frac{\Delta H{}^{\circ}}{\mathrm{RT}}+\frac{\Delta S{}^{\circ}}{R}$$

where Universal gas constant (8.314 J mol^−1^ K^−1^) is denoted by R; the Temperature in Kelvin (K) is denoted by T and Langmuir’s constant is denoted by b. Furthermore, the slope and intercept of the van’t Hoff plot (ln b vs. $\frac{1}{T}$), as shown in Figure 6, was used to calculate the values of ΔH° and ΔS°, respectively. The results obtained for the thermodynamic parameters are compiled in Table 3.

The positive value of ΔH° further confirmed that the adsorption process was endothermic in nature. Using the Langmuir constant, Gibbs free energy change, calculated at different temperatures, was in the range of −30.7 to −31.4 and −30.4 to −31.1 kJ mol^−1^ for Pb(II) and Cd(II), respectively. However, these values are higher in the case of Pb(II) as compared to Cd(II). Thus, it can be concluded that the adsorption process at different temperatures was spontaneous in nature and thermodynamically feasible. Moreover, the positive values of ΔS° obtained from the van’t Hoff plot for Pb(II) and Cd(II) indicate the affinity of the *PEFS* for the metals [45]. Hence, on the basis of thermodynamic studies, it can be concluded that the feasibility and spontaneity of the removal of Pb(II) are slightly more than Cd(II) on *PEFS*.

### 2.7. Biosorption Kinetics

**Figure 7:**
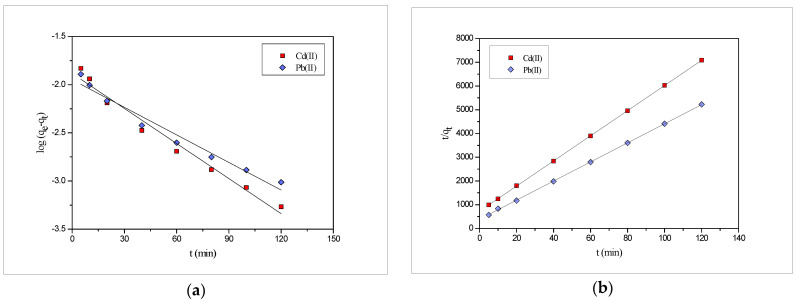
(**a**) Pseudo-first order; (**b**) pseudo-second order kinetic for the biosorption of Pb(II) and Cd(II) onto *PEFS* at 3 × 10^−5^ M.

The kinetic test for the biosorption of Pb(II) and Cd(II) at *PEFS* was carried out at different time intervals. The mechanism of the biosorption process was determined using different models [46,47,48] since different system conforms to different models. The kinetic model, such as pseudo-first order and pseudo-second order, were used to decipher the fitting to kinetic data. The Lagergren pseudo-first order kinetic equation [46], as mentioned below, was widely used:

(6) $${\log\;(q}_{e}-q_{t})={\mathrm{logq}}_{e}-\frac{k_{1}}{2.303}\;t$$

where q_e_ and q_t_ are the amounts adsorbed at equilibrium and time t, respectively, and k_1_ is the pseudo-first order rate constant. A plot was made between log (q_e_–q_t_) vs. time t for Pb(II) and Cd(II) and is shown in Figure 7. The kinetic parameters were obtained using this and are presented in Table 4.

Ho’s second order equation [49] also known as the pseudo-second order kinetic model, was also used for the study and expressed as:

(7) $$\frac{t}{q_{t}}=\frac{1}{K_{2}q_{e}^{2}}+\frac{t}{q_{e}}$$

where the amount adsorbed at time t and equilibrium was denoted by q_t_ and q_e_, respectively, and k_2_ is the pseudo-second order rate constant. A plot made between t/q_t_ vs. t was presented in Figure 7 and the value of k_2_ and q_e_ are determined from it. The results obtained were compiled in Table 4. Furthermore, it was observed that the value of the correlation coefficient (R^2^) in the case of the pseudo-second order kinetic model was higher than that of the pseudo-first order kinetic model, and the experimental value of the amount adsorbed at the equilibrium (q_e_) was in agreement with that of the calculated one obtained from the pseudo-second order model.

Moreover, the experimental amount adsorbed value (q_e(exp)_) was close to the calculated values (q_e(cal)_) of the pseudo-second order model, which confirms the fact that kinetic data in the present study fits well with the pseudo-second order kinetic model.

## 3. Materials and Methods

### 3.1. Chemicals

The chemicals used in the present work were of analytical grade. Pb(NO_3_)_2_ and Cd(NO_3_)_2_·4H_2_O were purchased from Merck Specialties Private Limited, Mumbai, India and HiMedia Mumbai, India, respectively. Further, double-distilled water was used to prepare the experimental solutions and dilutions.

### 3.2. Preparation of PEFS

The *PEFS* was purchased from a local vendor. Further, the procured *PEFS* was subjected to multiple washing steps to remove the dust particles using distilled water and then oven-dried for 24 h at 100 °C. The dried *PEFS* materials were crushed into fine particles using a grinder and sieved to obtain a uniform particle size biomaterial. The obtained homogenous-sized biomaterial was stored in an airtight container until further application.

### 3.3. Preparation of the Solutions

A stock solution of the Pb(II) and Cd(II), with a concentration of 1 × 10^−3^ M, was prepared by dissolving the desired amount of Pb(NO_3_)_2_ and Cd(NO_3_)_2_·4H_2_O, respectively, in double-distilled water. A series of experimental solutions were prepared by the dilution of the stock solutions.

### 3.4. Adsorption Studies

Batch biosorption methodology was used to elucidate the effect of contact time, initial metal ion concentration and solution pH on Pb(II) and Cd(II) biosorption at *PEFS*. For this, in the stoppered glass tubes, a weighed amount (0.01 g) of the *PEFS* was added to 10 mL of the Pb(II) and Cd(II)solution of varying concentrations. All the solutions were kept under the desired temperature and agitated until the achievement of equilibrium using a temperature-controlled shaker bath. Further, the atomic absorption spectrophotometer (ECIL, AAS-4129, Hyderabad, India) was used to measure the concentration of model pollutants, i.e., Pb(II) and Cd(II) metal ions.

The amount of metal adsorbed onto the *PEFS* was measured as the difference between the metal adsorbed on the *PEFS* and the metal ions present in the solution after adsorption using the below-mentioned equation:

(8) $$q_{e}=\frac{\left(C_{0}{-\;C}_{e}\right)V}{W}$$

where

q_e_ is the metal uptake (mol·g^−1^);

C_0_ and C_e_ are the initial and equilibrium concentrations (mol·L^−1^) in the solution, respectively,

V is the solution volume (L);

W is the mass (g) of the biosorbent used.

### 3.5. Characterisation of PEFS

The *PEFS* was characterized -in our previous work [37]. The inorganic elements in *PEFS* were investigated with the help of the inductively coupled plasma optical emission spectrometer (Perkin Elmer, model Optima 7000 DV, Waltham, MA, USA). An elemental analyser (Vario, Micro CHNS Analyser, Hesse, Germany) was used to study the weight percentages of carbon, hydrogen, nitrogen and sulfur in *PEFS*. The surface groups on *PEFS* were predicted with the help of Fourier transform infrared spectroscopy (FT-IR) by using the Perkin Elmer model Paragon 1000 PC spectrophotometer, Waltham, MA, USA. X-ray diffraction (XRD) patterns for *PEFS* were studied with an X-ray diffractometer Rigaku Smart Lab diffractometer, Tokyo, Japan. The surface morphology of *PEFS* was determined by field emission scanning electron microscopy (FE-SEM, Tescan Mira 3, Brno, Czech Republic).

## 4. Conclusions

The potential of *PEFS* has been tested for the sustainable remediation of noxious carcinogenic metal ions Pb(II) and Cd(II). Based on the results obtained, the following conclusions have been drawn:

1. The adsorption of Pb(II) and Cd(II) onto *PEFS* depends on the contact time, initial metal ion concentration, solution pH and temperature. The maximum amount (80%) of Pb(II) and Cd(II) ion was removed in less than 60 min and thereafter, equilibrium was achieved in 120 min.
2. The experimental sorption capacity for Pb(II) at pH 5 and Cd(II) at pH 6with contact time 180 min and a biosorbent amount of 0.01 g was found to be 0.048 and 0.026 mmol·g^−1^, respectively, at 25 °C.
3. Comparative results revealed that the removal of Pb(II) is more than that of Cd(II). The biosorption behavior of metal ions on the *PEFS* is a plausible mechanism with physical adsorption, surface complexation, electrostatic attraction and ion exchange as conventional pathways for explaining the removal of Pb(II) and Cd(II) by *PEFS*.
4. Kinetic data fits well with the pseudo-second order model, while the isotherm data fits well with both adsorption models, i.e., Langmuir and Freundlich.
5. The thermodynamic parameter shows that the biosorption process is spontaneous and favorable.
6. Keeping in view the results of the removal of Pb(II) and Cd(II), the biosorbent may be tried for wastewater/seawater containing these types of metal ions.

## Figures and Tables

**Table 1:** Langmuir and Freundlich isotherm constants and correlation coefficients for biosorption Pb(II) and Cd(II) onto *PEFS* at different temperatures.

Metals	Temp (°C)	Langmuir			Freundlich		
		q_max_ (mmol·g^−1^)	b(L·mol^−1^)	R^2^	K_f_ (mmol·g^−1^)	n	R^2^
Pb(II)	25	0.048	2.36 × 10^5^	0.980	8.90	2.03	0.980
	35	0.052	2.62 × 10^5^	0.984	9.54	2.05	0.984
	45	0.057	3.15 × 10^5^	0.974	10.20	2.08	0.981
Cd(II)	25	0.027	2.13 × 10^5^	0.996	8.71	1.85	0.990
	35	0.028	2.51 × 10^5^	0.992	9.37	1.87	0.991
	45	0.031	2.80 × 10^5^	0.987	9.92	1.88	0.988

**Table 2:** Characteristics properties of *PEFS* *.

Elemental Analysis of *PEFS*							
C%	N%	S%	H%				
46.5	0.07	0.14	6.2				
**Inorganic Amount in 1 kg**							
K (mg)	Na (mg)	Mg (mg)	Ca (mg)	Fe (mg)	P (mg)	Cu (mg)	Mn (mg)
432.2	253	101.3	860.14	26.7	267.99	5.4	0.81
**FTIR Analysis of *PEFS***							
O–H stretching	C–H stretching	C=O stretching (carboxyl, aldehyde, ketone and ester)	carboxylate anion stretching	stretching due to Methoxy group	stretching due to ether and epoxide	C–O stretching of alcohol	-OH bending
3448	3000–2800 cm^−1^	1740–1700 cm^−1^	1637 cm^−1^	1453 and 1423 cm^−1^	1254 cm^−1^	1044 cm^−1^	615 cm^−1^
**XRD-analysis of *PEFS***							
2θ = 16° and 22° Corresponding to cellulose							
**N_2_ adsorption isotherms**							
Low surface area and non-porous							
**SEM analysis**							
Compact surface structure							

* [37].

**Table 3:** Thermodynamic parameters for biosorption of Pb(II) and Cd(II) onto *PEFS*.

Metals	Temperature (°C)	−ΔG° (kJ·mol^−1^)	ΔS° (J·mol^−1^·K^−1^)	ΔH° (kJ·mol^−1^)
Pb(II)	25	30.7		
	35	30.9	141	11.3
	45	31.4		
Cd(II)	25	30.4		
	35	30.8	138	10.8
	45	31.1		

**Table 4:** Kinetic parameters for the biosorption of Pb(II) and Cd(II) onto *PEFS*.

Metal	C_o_ (mol·L^−1^)	q_e (exp)_ (mmol·g^−1^)	Pseudo-First Order			Pseudo-Second Order		
			q_e_ (mmol·g^−1^)	K_1_ (min^−1^)	R^2^	q_e_ (mmol·g^−1^)	K_2_ (g·mmol^−1^·min^−1^)	R^2^
Pb(II)	3 × 10^−5^	0.024	0.013	0.019	0.743	0.025	4.19	0.998
Cd(II)	3 × 10^−5^	0.018	0.011	0.022	0.969	0.019	3.88	0.999

## Data Availability

Not applicable.
